# Interleaved premotor rTMS does not affect motor sequence learning

**DOI:** 10.1038/s41598-026-60507-9

**Published:** 2026-07-16

**Authors:** Felix Psurek, Malte Tiburtius, Joseph Classen, Gesa Hartwigsen, Jost-Julian Rumpf

**Affiliations:** 1 Department of Neurology, University of Leipzig Medical Center, Leipzig, Germany; 2 Wilhelm Wundt Institute for Psychology, Leipzig, Germany; 3 Max Planck Institute for Human Cognitive and Brain Sciences, Leipzig, Germany

**Keywords:** Neuroscience, Physiology

## Abstract

Acquiring new motor skills is a vital component of lifelong learning and underpins everyday activities. Performance improvements through training evolve from an initially fragile state to one that becomes increasingly resistant to disruption, a process known as motor consolidation. Recent research has indicated that learning takes place even during brief periods of rest – termed micro-offline learning. In healthy young individuals, such intervals may represent the primary source of performance enhancements during training sessions. Other studies have identified both the primary motor cortex (M1) and the premotor cortex (PMC) as pivotal neural structures for motor consolidation. Specifically, it has been demonstrated that 10 Hz interleaved repetitive transcranial magnetic stimulation (i-rTMS) to M1 during rest intervals between active motor training blocks can enhance post-training offline consolidation without influencing performance during training. Here, we examined whether 10 Hz i-rTMS to the PMC produces similar effects. Contrary to our expectations, our results suggest that premotor i-rTMS does not affect online or offline learning, at both microscopic and macroscopic temporal scales. We also measured corticospinal excitability before and after training, but no detectable effects of training or i-rTMS were observed. Taken together, these findings provide no evidence for an effect of i-rTMS of the PMC on online or offline motor sequence learning. We speculate that consolidation during offline processing in short rest periods between active training phases may not necessarily require involvement of the premotor cortex.

## Introduction

The acquisition of new motor skills is a dynamic, multistage process that unfolds both ‘online’, during active movement execution, and ‘offline’, during rest intervals between training sessions^1^. This latter phase, termed offline motor memory consolidation, serves to stabilize the initially fragile, training-induced skill-model, transforming it into a robust memory representation that is resistant to interference in the absence of further practice^2–4^.

Sequential motor skill learning depends on dynamic interactions among a distributed neural network, including the primary and secondary motor cortices, parietal regions, basal ganglia, cerebellum, hippocampus, and spinal circuitry^5–10^. Within this architecture, the dorsal premotor cortex (PMC) has emerged as a critical node for integrating spatial and temporal movement features. The PMC plays a pivotal role during online learning, coordinating the translation of sequence knowledge - retrieved from a distributed network including hippocampus, parietal areas, and striatum - into spatially organized motor commands for specific effectors (i.e., the fingers)^11,12^. Through its dense reciprocal projections to M1, the PMC is also believed to modulate corticospinal excitability and synaptic plasticity^13–16^, facilitating the formation of motor memories during practice and potentially gating their subsequent offline consolidation^17^.

Evidence for this consolidation role is derived from non-invasive brain stimulation (NIBS) and imaging studies^16,17,19,20^. Previous work by Tunovic et al.^18^ demonstrated a significant relationship between post-training corticospinal excitability and the magnitude of consolidation: specifically, preventing the typical post-training decrease of M1 excitability to baseline levels facilitated offline skill improvements. Several studies have shown that stimulation of the dorsal premotor cortex can indeed modulate these M1 excitability states, including our own work where we observed that post-training inhibition of the PMC with 1 Hz rTMS leads to an increase in M1 excitability^37^. Collectively, these findings suggest that PMC stimulation can modulate M1 excitability and may directly influence consolidation. However, interpreting previous results is complicated by substantial methodological heterogeneity regarding stimulation protocols (e.g., “excitatory” vs. “inhibitory”) and timing (pre-, peri-, or post-training), which has led to variable behavioral outcomes^19,20^.

Understanding the exact role of PMC in motor learning is further complicated by the distinction between explicit and implicit learning mechanisms. While the dorsal PMC is a critical node for both types of learning, it plays a distinct role in explicit motor sequence acquisition, serving as a key interface where declarative knowledge of the movement pattern is translated into motor output^17,21^. Conversely, implicit learning is thought to rely more heavily on plasticity within M1, the supplementary motor area, and striatal loops^22–25^. This functional dissociation implies that the PMC’s contribution to consolidation is likely context-dependent, acting as a bridge between cognitive control networks and motor pathways specifically when performance is guided by explicit knowledge^17^.

More recently, a paradigm shift has emphasized that most early performance improvements occur not primarily during active practice but predominantly, during the short rest periods between practice blocks—a phenomenon termed “micro-offline learning” or rapid consolidation^26^. In contrast, performance during active practice blocks (“micro-online learning”) often shows decrements, such that the net learning effect results primarily from positive micro-offline gains outweighing negative micro-online changes.

In previous work, our group has shown that 10 Hz interleaved rTMS (i-rTMS) over M1 during the short rest intervals between practice blocks enhanced subsequent consolidation^27^.

In the present study, we investigated the role of the premotor cortex in micro-online/micro-offline learning and in post-training offline motor memory consolidation. Our hypothesis relies on evidence suggesting a functional dissociation between M1 and PMC during the consolidation of motor skills. While 10 Hz i-rTMS of M1 has been shown to facilitate consolidation^27^, previous work on implicit learning indicates that the PMC and M1 may play opposing roles during offline processing^20^. Specifically, increased excitability in the dorsal premotor cortex has been associated with an inhibition of consolidation processes in M1^20^. Consequently, we hypothesized that increasing PMC excitability during rest intervals via 10 Hz i-rTMS would interfere with this delicate balance and impair subsequent offline consolidation.

Behavioral stimulation effects were assessed using a delayed retest seven hours after interleaved PMC-rTMS. In addition, we measured stimulation-induced changes in CSE to examine potential interactions between PMC and M1 during the training session (including effects on micro-online and micro-offline learning) and post-training consolidation and test the proposed relationship between post-training CSE modulation and offline consolidation^18^. If consolidation processes are indeed malleable through stimulation of the PMC, this could open new avenues for developing NIBS-based interventions aimed at optimizing motor memory consolidation in clinical and rehabilitative contexts.

## Methods

### Participants

Forty healthy, right-handed young adults were recruited via the local network of Leipzig University. None of the participants had a history of neurological or psychiatric disorders, nor any alcohol or substance abuse. To ensure comparability, individuals with professional musical training or other specialized skills that could potentially influence the outcome of the motor sequence learning task—such as professional typists—were excluded from participation.

Prior to the study, all participants completed a screening questionnaire to assess eligibility for transcranial magnetic stimulation (TMS), addressing factors such as pregnancy and use of centrally acting medications. Depressive symptoms were screened using the short version of the Beck Depression Inventory^29^. Current alertness was assessed both in the morning before training and in the afternoon prior to retesting using the Stanford Sleepiness Scale^30^.

All participants were TMS-naïve and had no prior experience with the motor sequence learning task. Written informed consent was obtained from all subjects prior to participation. The study was approved by the local ethics committee of the Medical Faculty at Leipzig University and conducted in accordance with the Declaration of Helsinki.

Participants were randomly assigned to one of two groups: 20 received sham stimulation, while the other 20 underwent effective stimulation over the dorsal premotor cortex (PMC). The experiment consisted of two sessions—one in the morning (between 8:00 and 11:00 AM) and a second session approximately seven hours after the start of the first session.

### Experimental design

**Fig. 1:**
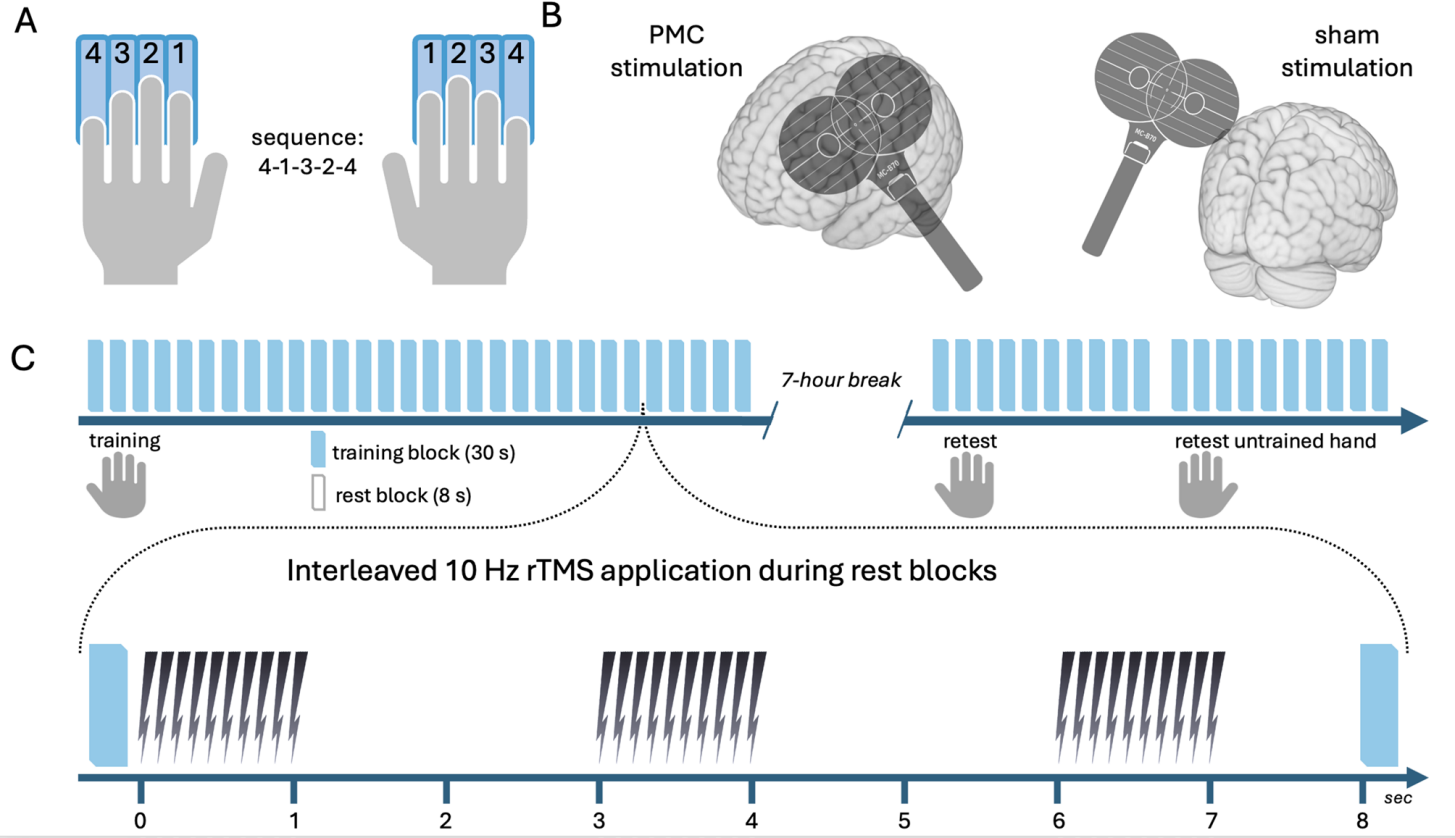
Experimental design. (**A**) Schematic representation of the explicit finger tapping task, illustrating the sequence of key presses and the corresponding finger assignments for both the right and left hand on the keyboard. (**B**) Illustration of the presumed TMS sites for the sham and dorsal premotor cortex (PMC) conditions. For sham stimulation, the peak magnetic field was directed away from the scalp, ensuring that no effective cortical stimulation occurred. (**C**) Overview of the experimental procedure: Following 30 training blocks performed with the right hand, participants had a 7-hour break. This was followed by a retest of the right hand and subsequently a retest of the left hand, each comprising 10 blocks. During the 8-second pauses between training blocks, interleaved 10 Hz rTMS was administered at seconds 1, 4, and 7.

All participants performed the same motor sequence learning task - a modified version of the explicit sequential finger tapping paradigm described by Karni et al.^10^. The task was executed on a keyboard using the right (dominant) hand. Each sequence comprised five key presses in the order 4–1–3–2–4, corresponding to the little finger (4), index finger (1), ring finger (3), and middle finger (2) (Fig. 1A). Prior to the main training phase, participants completed a brief verification sequence and a practice block containing three correctly executed sequences. The training phase consisted of 30 blocks, each separated by an 8-second pause. Each block comprised six sequences, amounting to a total of 30 key presses per block. Blocks concluded automatically after 30 key presses, regardless of accuracy. Block onset was indicated by a green cross on a black background, while completion was signaled by a red cross.

Participants were instructed to execute the sequence as quickly and accurately as possible. Following a break of approximately seven hours, a retest was administered, comprising 10 blocks of the same sequence. The retest was administered first using the right hand, followed by the left hand. For the left hand, the sequence and key mapping remained identical 4–1–3–2–4 such that, for example, the number 4 corresponded to the little finger, while 1 referred to the index finger. The primary outcome measures were correct sequence duration (CSD), defined as the time per correctly executed sequence, and accuracy, defined as the number of correct sequences per block (maximum 6 per block).

Micro-online learning was operationally defined as the temporal difference between the first and last sequence within a given block, whereas micro-offline learning was characterized as the difference between the last sequence preceding a pause and the first sequence of the subsequent block following the pause. Sign conventions were adopted such that positive values represented improvements in correct sequence duration (CSD), thereby reflecting increased speed. In accordance with previous studies and to facilitate more informative graphical representation, micro-online and micro-offline gains were depicted as continuous cumulative sums across the training session^28^. This approach enabled clear visualization of the cumulative contributions of micro-offline and micro-online gains to overall performance improvements by the end of training.

### Interleaved rTMS

Following informed consent and completion of questionnaires, the motor hotspot for the right hand was localized using transcranial magnetic stimulation (TMS). The hotspot for the abductor pollicis brevis (APB) muscle of the right hand was identified by tangential placement of the coil over the left hemisphere. Motor-evoked potentials (MEPs) were recorded using EMG (Digitimer D360, Digitimer Ltd., Letchworth Garden, UK). The coil handle was oriented at approximately 45° in a posterior-inferior direction. After identifying and marking the motor cortex (M1) in the neuronavigation system, individual resting motor threshold (RMT) was determined using an adaptive Parameter Estimation by Sequential Testing (PEST) procedure. This algorithm adjusted the stimulation intensity stepwise to estimate the threshold at which a motor response was elicited with a probability of 50%.

To assess baseline cortical excitability, 20 single-pulse MEPs were elicited at an interstimulus interval of 3 s and an intensity of 120% RMT. This measurement was performed immediately before and directly after completion of the motor sequence learning task to capture changes in corticospinal excitability (CSE) that might have resulted from the learning task and/or the stimulation.

For stimulation of the PMC, the coil was positioned 25 mm anterior to the individual M1 hotspot^13^ (Fig. 1B). A neuronavigation system (Brainsight 2, Rogue Research, Canada) was used to ensure stable and consistent positioning of the TMS coil over the identified M1 and PMC stimulation sites throughout the experiment via real-time visual feedback to the experimenter on a generic template brain. We did not use individual MRIs for target localization. In the sham stimulation condition, the coil was rotated 90° away from the scalp, so that the underside of the coil faced outward into the room while the outer rim continued to touch the head. This approach was chosen to preserve the characteristic sensory features of TMS while avoiding the additional cutaneous afferent activation associated with electrically active sham coils.

Interleaved repetitive TMS (i-rTMS) was administered during the 8-second breaks between training blocks. During each pause, 10 pulses at 10 Hz and 90% RMT were delivered at the 0th, 3rd, and 6th seconds, with each train separated by 2.1 s. The total stimulation duration per block was therefore 900 ms, precisely matching the stimulation protocol used in our group’s previous study^27^ (Fig. 1C).

TMS was administered with a MagVenture MC-B70 70 mm figure-of-eight coil in combination with a MagPro X100 stimulator (MagVenture, Farum, Denmark).

### Data analysis

The effects of repeated practice on task performance throughout the initial training and delayed retest sessions were examined using separate repeated measures analyses of variance (rmANOVA) for speed, accuracy, and micro-offline/online learning. The analyses included *Group* (PMC, SHAM i-rTMS) as the between-subject factor and *Block* (e.g., B1 to B14) as the within-subject factor. This design permitted evaluation of potential between-group differences in both the magnitude and rate of performance changes resulting from repeated practice during training and retest sessions. Offline consolidation effects were calculated as the difference between each participant’s end-of-training baseline (EoT; defined as the average correct sequence duration across the final four blocks of the training session) and performance at the onset of the delayed retest (SoRR for the right hand, SoRL for the left hand; defined as the first four blocks of the delayed retest). Positive values indicated offline performance gains relative to EoT, whereas negative values denoted offline performance decrements (i.e., speed loss).

Electromyographic data were recorded using CED Signal (Cambridge Electronic Design Ltd., Cambridge, England) and subsequently processed manually with the palMEP tool^32^. All recorded motor-evoked potentials (MEPs) were inspected individually and excluded if artefacts, such as pre-activation or voluntary movements, were detected in the EMG traces. To assess potential rTMS-induced effects on corticospinal excitability (CSE), MEP amplitude values were averaged separately for pre- and post-assessment time points (MEPpre and MEPpost) and analyzed using a rmANOVA, with Time (MEPpre, MEPpost) as the within-subject factor and Group as the between-subjects factor.

The experimental task was programmed in MATLAB (MathWorks, Natick, USA), which was also used to externally trigger TMS stimulation. Parameters were extracted from the raw data using custom MATLAB scripts. All statistical analyses were conducted using jamovi^33,34^. Normality of the data distribution was assessed using the Shapiro–Wilk test. An alpha level of *p* < 0.05 was applied to all statistical tests. Repeated measures ANOVAs (rmANOVAs) were evaluated for violations of sphericity; if necessary, the degrees of freedom and p-values were adjusted using the Greenhouse–Geisser correction. Spearman’s rank correlation coefficient was employed to assess associations and the magnitude of offline performance changes.

## Results

No significant demographic differences were observed between participants in the PMC and SHAM groups. Age was comparable across groups (mean age = 23.9 years, SD = 4.08; *p* = 0.302), as was handedness according to the Edinburgh Handedness Inventory^31^ (mean = 77.0, SD = 18.6). Sex distribution did not differ significantly (*p* = 0.752; PMC: 13 women, SHAM: 12 women)

**Fig. 2:**
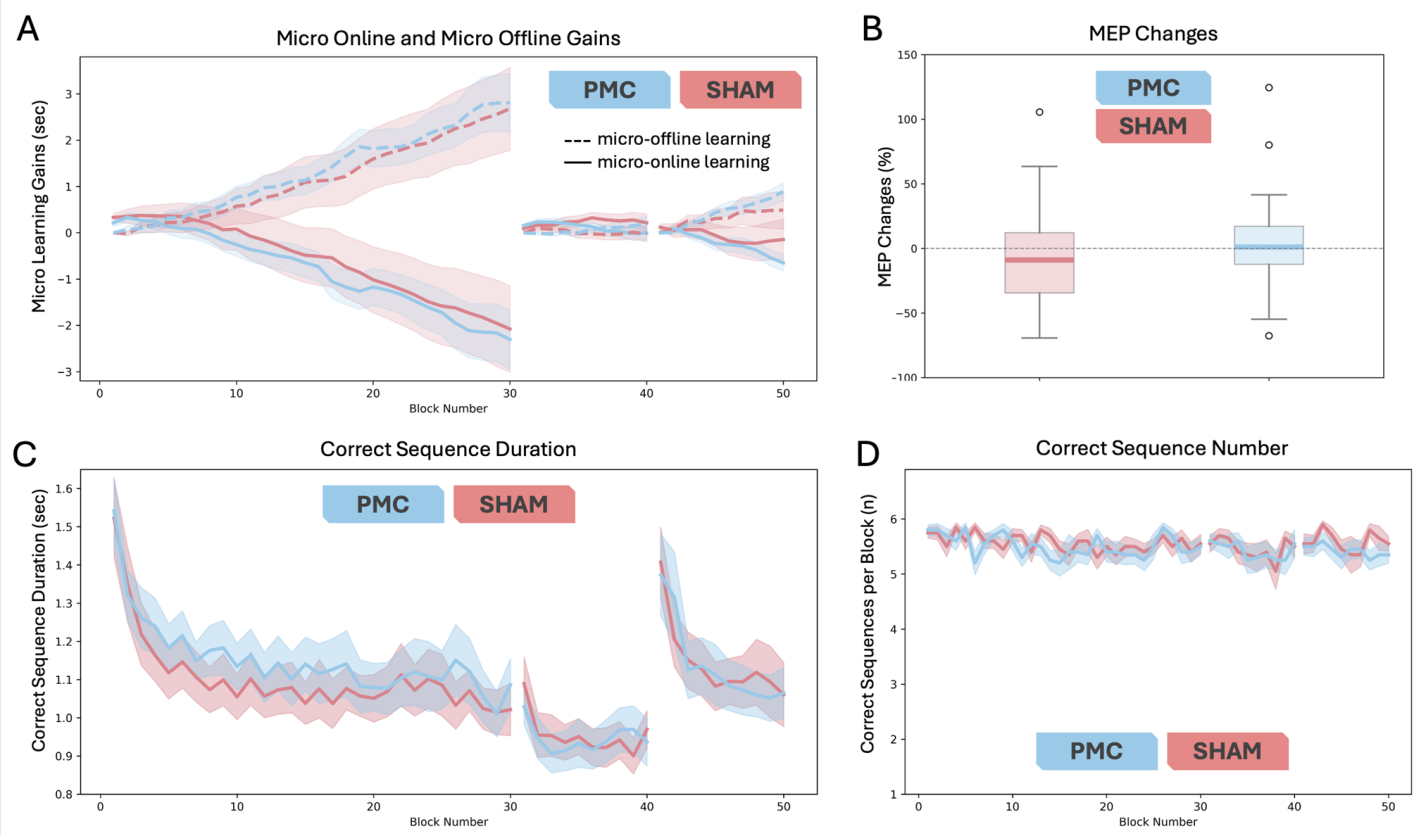
Illustration of Selected Results. Blocks 1–30 correspond to the morning training session, blocks 31–40 represent the right-hand retest administered after a 7-hour interval, and blocks 41–50 correlate with the left-hand retest. (**A**) Cumulative micro-online and micro-offline gains across training and retest blocks, with dashed lines indicating micro-offline learning and solid lines representing micro-online learning. Positive values denote improvements in speed. (**B**) Relative changes in motor evoked potentials before and after the morning training are presented as suggestive markers for corticospinal excitability, with interquartile ranges shown. (**C**) Correct sequence duration (in seconds) for each block. (**D**) The number of correct sequences per block (maximum 6 per 30 key presses) is displayed. For panels (**A**), (**C**), and (**D**), error bars represent the standard error of the mean (SEM).

Participants demonstrated significant learning during active training, as evidenced by a reduction in correct sequence duration (CSD) over the 30 blocks. A repeated measures ANOVA revealed a significant effect of *Block* (F(7.41, 274.00) = 17.93, *p* < 0.001). There was no significant difference in performance between groups (between-subjects effect of *Group*: F(1,37)= 0.298, *p* = 0.588), nor was there a significant *Block × Group* interaction (F(7.41, 274.00) = 0.524, *p* = 0.825). Both groups achieved similar performance levels by the end of training, with no statistically significant difference (30th block CSD: SHAM mean = 1.02 s, SD = 0.302; PMC mean = 1.09 s, SD = 0.302) (Fig. 2).

The delayed retest conducted in the afternoon also revealed significant improvements across the 10 blocks for the trained right hand as indicated by a main effect of *Block* (F(5.52, 209.74) = 6.63, *p* < 0.001), with no significant *Block × Group* interaction (F(5.52, 209.74) = 1.47, *p* = 0.195). A similar pattern emerged for the left hand, demonstrating a significant main effect of *Block* (F(2.94, 108.66) = 24.62, *p* < 0.001) in the absence of a *Block × Group* interaction (F(2.94, 108.66) = 0.982, *p* = 0.403).

In summary, macro-online training was characterized by a significant training effect during both the active training phase and the delayed active retest sessions for the right and left hand, respectively. This was evidenced by improved performance, reflected in reduced correct sequence duration across all sessions (training, right-hand retest, left-hand retest).

With respect to macro-offline learning, we compared the training plateau ‘End of Training’ (EoT) with the ‘Start of Retest right’ (SoRR). A repeated measures ANOVA revealed a significant effect of *Session* (F(1,38)= 12.36, *p* = 0.001), with a mean macro-offline performance gain evidenced by an improvement in speed of 0.0846 ± 0.0240 s. The site of stimulation, did not exert a significant impact on the offline consolidation of the right hand, as indicated by the absence of a *Session* × *Group* interaction (F(1,38)= 2.13, *p* = 0.153).

A similar analytical approach was adopted for the left hand, comparing the first four blocks of the left-hand retest (“Start of Retest left”, SoRL) with the EoT. Here, a significant effect of *Session* again was observed (F(1,38)= 26.39, *p* < 0.001), without evidence of a *Session* × *Group* interaction (F(1,38)= 0.09, *p* = 0.770). The left hand demonstrated a mean slowing of -0.180 ± 0.0350 s compared to the EoT for the right hand.

To assess the intermanual transfer of learning gains, we compared performance of the untrained (left) hand at retest with the initial baseline performance of the trained (right) hand. Notably, participants performance with the sequence-naïve left hand surpassed the initial performance level of the dominant right hand, indicating a substantial transfer of the consolidated motor skill. A rmANOVA revealed a significant effect of *Session* (F(1,38)= 6.944, *p* = 0.012), with no significant *Session* × *Group* interaction (F(1,38)= 0.243, *p* = 0.625), suggesting that rTMS applied to the PMC did not modulate this transfer effect.

We observed a significant micro-online process during the training blocks, as evidenced by a main effect of *Block* (F(1.47, 55.80) = 19.80, *p* < 0.001). Descriptive statistics of cumulative micro-online gains revealed that, interestingly, positive values—indicative of improvements in speed per correctly executed key sequence—were only present during the initial eight blocks of training. In contrast, blocks 9–30 consistently exhibited negative values, reflecting performance decrements during active key sequence execution within each block, a finding that aligns with previous reports in young, healthy participants.

During the right-hand retest, micro-online learning did not result in significant changes in correct sequence duration (effect of *Block*: F(2.05, 77,71) = 0.835 *p* = 0.440). As anticipated, given the absence of prior active training, significant micro-online learning was observed for the left hand during its retest (effect of *Block*: F(1.46, 55.58) = 3.89, *p* = 0.038). Notably, participants reached the “switch” point—where the sign of performance change reversed—after just the third block in the left-hand retest. From block 4 to 10 of the left-hand retest, active performance again deteriorated within blocks, as indicated by negative values.

The pattern for micro-offline learning—i.e., performance gains during the brief inter-block pauses, which also coincided with the application of PMC rTMS or sham stimulation—differed. Descriptive statistics indicated consistently mean positive performance improvements both across all pauses during training and during retests. A repeated measures ANOVA revealed a significant effect of *Block* for both the training phase and the left-hand retest (F(1.52, 57.91) = 23.50, *p* < 0.001; F(1.46, 55.47) = 6.809, *p* = 0.005). Similarly, positive micro-offline gains were also observed in the right-hand retest; however, these did not reach statistical significance (F(1.93, 73.33) = 0.40, *p* = 0.663).

The i-rTMS intervention had no significant impact on micro-learning. Neither micro-online nor micro-offline learning exhibited a significant *Block × Group* interaction (micro-online: F(1.46, 55.30) = 0.10, *p* = 0.848; micro-offline: F(1.52, 57.91) = 0.10, *p* = 0.855).

With respect to accuracy, operationalized as the number of correct sequences per block (maximum of 6), participants consistently exhibited very high levels of accuracy throughout the experimental sessions. No significant main effects of *Block* were observed during training (F(14.5, 549.6) = 1.40, *p* = 0.143), right-hand retest (F(6.38, 242.49) = 1.51, *p* = 0.172), or left-hand retest (F(6.15, 233.80) = 0.815, *p* = 0.562). Furthermore, there was no statistically significant *Block × Group* interaction for accuracy during training (F(14.5, 549.6) = 1.14, *p* = 0.319), right-hand retest (F(6.38, 242.49) = 0.523, *p* = 0.801), or left-hand retest (F(6.15, 233.80) = 0.679, *p* = 0.670).

On average, the motor-evoked potentials measured prior to training across all participants were 1.21 mV ± 0.276 mV (SD), with no significant difference in baseline corticospinal excitability (CSE) observed between the groups (*p* = 0.294). Furthermore, there was no significant change in CSE during the training phase (main effect of *Time*: F(1,38) = 0.40, *p* = 0.531, mean CSE-Change = -0.0424 mV), and this change could not be attributed to the stimulation site, as indicated by a non-significant *Time × Group* interaction (F(1,38) = 0.163, *p* = 0.689).

No significant correlation was observed between changes in corticospinal excitability (CSE) and the magnitude of macro-offline performance gains, with Spearman’s rho values of -0.182 (*p* = 0.261) for the right hand and − 0.150 (*p* = 0.353) for the left hand. Consistent with typical task-related findings, we identified a significant correlation between correct sequence duration at the end of training and the extent of offline consolidation for the right hand. This indicates that participants that were slower at the end of the training phase exhibited relatively greater offline performance gains (Spearman’s rho = 0.470, *p* = 0.002).

## Discussion

The present study investigated the causal contribution of the dorsal premotor cortex (PMC) to explicit motor sequence learning, focusing on the temporal dynamics of skill acquisition during initial training (micro-learning) and retention at delayed retesting (macro-consolidation). By interleaving 10 Hz repetitive transcranial magnetic stimulation (i-rTMS) during brief rest intervals, we tested whether PMC engagement is critical for stabilizing explicit motor memories. Importantly, performance changes during brief rest intervals (“micro-offline learning”) may reflect either rapid intrinsic motor memory consolidation processes or, alternatively, transient recovery from reactive inhibition and performance-related fatigue accumulated during practice. Contrary to our hypothesis, PMC modulation left behavioral performance unaffected across all temporal scales: it did not alter the micro-online losses during active training blocks, nor did it modulate micro-offline gains accumulated during short breaks within the initial training session, or the long-term macro-offline consolidation observed at the delayed retest. Furthermore, we found no evidence of stimulation-induced alterations in corticospinal excitability, nor any correlation between changes in corticospinal excitability and behavioral outcomes. Despite the absence of significant stimulation effects, our data reveal two robust behavioral phenomena: the replication of micro-learning dynamics in an explicit task and a remarkable, effector-independent intermanual transfer of the acquired skill.

A central finding of this study is that micro-offline learning—the rapid performance gains observed after short rest intervals—proceeds independently of PMC modulation. In our cohort, participants exhibited the characteristic pattern previously described for young individuals^26,28^: a progressive performance decrement during continuous practice (micro-online ‘fatigue’) followed by a rebound during rest.

Recent evidence challenges the concept of micro consolidation, suggesting that micro-offline improvements may primarily reflect the dissipation of temporary inhibition in motor outputs rather than the transformation of the memory trace itself^35,36^. If micro-offline gains in explicit tasks primarily reflect recovery from reactive inhibition and performance-related fatigue, including the washout of transient inhibition within M1, the PMC may play a negligible role during the rest phase. Our null result could support this “fatigue-recovery” hypothesis for the premotor cortex: while the PMC is crucial for online sequence planning, it does not appear to be the area where the restorative processes of the rest interval take place.

Alternatively, if true rapid motor memory consolidation does occur, recent work by Mylonas et al.^28^ suggests it may be driven by hippocampal rather than premotor mechanisms. In this framework, the hippocampus binds temporal sequence elements during rest, while M1 executes the motor plan. The PMC, serving as an intermediate node, may thus be bypassed during these specific offline windows in explicit learning, explaining its resistance to rTMS interference.

We further observed no measurable effects of PMC i-rTMS on macro-offline consolidation assessed at the delayed retest. Both groups showed significant retention and even slight offline gains, but this occurred independently of the intervention. This dissociation contrasts with studies on implicit learning, where PMC stimulation has been shown to modulate offline stabilization^17,20^. Consequently, the consolidation of explicit skills may not depend on the PMC during the early post-training phase, or at least not to a degree that can be disrupted by the used i-rTMS protocol.

Previous work proposed that post-training increases in CSE serve as a biomarker for subsequent successful offline consolidation at the macro-level^18^. By contrast, our findings—alongside those from a recent investigation conducted in our laboratory^37^—demonstrate clear behavioral evidence of learning and post-training consolidation, despite the absence of significant group-level changes in corticospinal excitability. Moreover, individual variations in CSE did not predict behavioral retention. It is likely that CSE changes reflect state-dependent M1 excitability rather than indicating motor sequence memory formation in M1. This may be particularly the case in explicit tasks where the “skill” is partly encoded in upstream cognitive structures (e.g. PMC, SMA, DLPFC) rather than solely in synaptic weights of the corticospinal tract.

One remarkable result of our study is the magnitude of intermanual transfer. At the delayed retest, the untrained left hand performed the sequence not just better than baseline, but at a level comparable to the trained right hand. To our understanding, this indicates that the participants acquired a high-level, effector-independent representation of the sequence (e.g., “allocentric”) rather than a muscle-specific motor memory.

The observation that PMC stimulation failed to disrupt this transfer is particularly informative. The PMC – in addition to SMA, cingulate motor areas, posterior parietal cortex, basal ganglia and M1 - is classically considered a hub for interhemispheric transfer^11,17^. However, the preservation of transfer capacity in our PMC-stimulated group suggests that the abstract, explicit sequence knowledge is likely maintained by bilateral prefrontal or parietal networks that remain functionally intact despite PMC interference.

We acknowledge that a key limitation of the present study is the absence of physiological or behavioral evidence confirming effective modulation of premotor–motor circuitry; accordingly, the null findings should be interpreted with caution, as they may reflect insufficient neuromodulatory efficacy of the stimulation protocol rather than functional quiescence of the premotor cortex. However, we utilized precise neuronavigation and employed a stimulation protocol that has previously been shown to produce behaviorally relevant interactions with M1^27^. We furthermore consider our stimulation intensity sufficient to induce physiological effects, as previous studies have demonstrated robust rTMS effects at 90% RMT across a range of tasks and cortical regions^38–40^, as well as modulation of premotor–motor interactions using even lower subthreshold stimulation intensities such as 80–90% active motor threshold^13,14^. The consistency of our null results across multiple measures (micro-learning, macro-consolidation, CSE) points towards a true physiological dissociation rather than a methodological failure.

In summary, our findings suggest that explicit motor sequence learning is remarkably resilient to PMC modulation during rest intervals. Neither the rapid performance gains observed during training (micro-offline learning) nor the stabilization of memory over time (macro-offline consolidation) appear to depend on dorsal premotor excitability. Instead, our results are best interpreted as suggesting that micro-offline gains in explicit tasks may reflect recovery from fatigue (reactive inhibition) or hippocampal binding processes, whereas long-term retention and intermanual transfer are supported by a distributed cognitive network. Taken together, these findings point to a task-specific hierarchy in which the PMC contributes to online execution but does not appear to serve as the principal mechanism underlying the offline stabilization of explicit skills.

## Data Availability

The data that support the findings of this study are available from the corresponding author FP upon reasonable request.
